# Study Protocol for a Controlled Trial of Nutrition Education Intervention about Celiac Disease in Primary School: ZELIAKIDE Project

**DOI:** 10.3390/nu16030338

**Published:** 2024-01-23

**Authors:** Maialen Vázquez-Polo, Itziar Churruca, Gesala Perez-Junkera, Idoia Larretxi, Arrate Lasa, Jon Esparta, Leire Cantero-Ruiz de Eguino, Virginia Navarro

**Affiliations:** 1 Gluten 3S Research Group, Department of Nutrition and Food Science, Faculty of Pharmacy, University of the Basque Country UPV/EHU, 01006 Vitoria-Gasteiz, Spain; maialen.vazquez@ehu.eus (M.V.-P.); gesala.perez@ehu.eus (G.P.-J.); idoia.larrechi@ehu.eus (I.L.); arrate.lasa@ehu.eus (A.L.); jon.esparta@ehu.eus (J.E.); leire.canteror@ehu.eus (L.C.-R.d.E.); virginia.navarros@ehu.eus (V.N.); 2 Bioaraba, Nutrition and Food Safety Group, 01009 Vitoria-Gasteiz, Spain; 3 Centro Integral de Atención a Mayores San Prudencio, 01006 Vitoria-Gasteiz, Spain

**Keywords:** children, balanced diet, nutrition education, celiac disease, competence based learning, STEAM

## Abstract

The only treatment for celiac disease (CD) is a strict and lifelong gluten-free diet (GFD), which must be safe and nutritionally balanced. Avoiding gluten brings difficulties with following the diet and can affect the social life of people with CD. The Zeliakide Project is a nutrition education program aimed at increasing the knowledge of the general population about healthy diets, CD and GFD, and, therefore, to improve the social inclusion and quality of life of people with CD. It is a one-month intervention program, two-armed cluster, non-randomised and controlled trial, conducted among 10–12-year-old children. Pre- and post-intervention evaluation and 1 month follow-up will be carried out to assess the effectiveness of the program. It is based on competencies and their respective learning outcomes. The teaching methodology chosen is a STEAM methodology: inquiry-based learning (IBL). A teaching unit has been created to develop the project, which, in the future, will be useful for the self-application of the program. This study will provide a valid and useful tool to achieve changes in the diet at the school level and will help to promote the social inclusion of people with CD. Moreover, it will enforce the STEAM competences of children.

## 1. Introduction

Celiac disease (CD) is a systemic immune-mediated disorder caused by the ingestion of gluten and related prolamins in genetically predisposed individuals [1]. Its prevalence has increased significantly in the last 20 years [2,3] and it is not a well-understood disorder. In fact, this disease is sometimes undetected, with a 1:3-to-1:5 ratio between diagnosed and undiagnosed [4]. However, in most parts of the Western world, it affects approximately 1–2% of the population [5], even if wide differences have been found among countries and even regions [2,5,6].

Currently, the only treatment for the disease is a strict and lifelong gluten-free diet (GFD). Although the importance of the absence of gluten in the diet is obvious, the diet must remain nutritionally balanced in order to achieve an optimal health condition. Achieving a balanced diet is difficult for the general population. In the case of children and adolescents, for example, breakfasts and mid-morning snacks (very common in Spain) differ from the prototype of a healthy diet due to the excessive consumption of sweet foods [7,8,9]. Achieving a healthy diet is even more difficult for celiac patients. 

The GFD involves excluding certain foods from the diet, which can lead to nutritional deficiencies. In the Basque Autonomous Community (Northern Spain), we studied a cohort of patients with CD in a previous study and found that their diet was deficient in dietary fibre and complex carbohydrates and high in protein and saturated fats [7,8]. Similar results were found by other authors [9,10,11]. It should be noted that these dietary errors are also common in the general population. Even if guidelines for the general population are published frequently, specific guidelines for the celiac population (following restrictive diet) are much rarer [7,8]. 

In addition to guidelines, educational activities are necessary for the management of chronic diseases and represent one of the most recommended measures to address diseases, including CD [12]. These types of educational interventions rely on the competence of health professionals, and desirably, health professionals specialised in the area of education. 

The aim of these activities is to empower people suffering from the pathology and/or people close to the patient in the treatment of the disease, in order to maintain or improve their quality of life [12]. On the other hand, the main objective of education is to provide information, share practical knowledge and change behaviour, when needed. 

This educational process is fundamental in the CD, because apart from physical health, GFD greatly influences the quality of life of patients, especially at the social and family level [13]. One of the biggest handicaps is eating out, as it is difficult to find gluten-free dishes and to ensure that there has been no gluten cross-contact [14]. In addition, they feel socially excluded when they have to check their food offer or even refuse it when they are eating out because of their situation. Moreover, a study by Zarkadas et al. found that 80% of the members of a celiac association in Canada avoided eating out at restaurants [15]. Another study of the same group found that restaurant menus are limited and eating away from home remains problematic, even if the situation is slowly improving [16]. 

In any case, strict GFD is fundamental for the physical health of people with CD, but also for their mental health. In fact, Halmos et al. linked a lower risk of psychological distress to a higher adherence to the GFD, which is very important for a good quality of life [17]. In this line, some studies have reported that health education needs to be developed and provided to people with CD in order to enable them to cope with their situation [18,19]. However, mental health is also closely related to relationships, especially during childhood. Barrio et al. observed that one of the biggest concerns of Spanish children with celiac disease was the relationship with their friends [20]. In addition to impacting individuals who have celiac disease (CD), the condition also has an impact on the quality of life of those in their immediate circle. This is due to the influence of the disease on everyday activities, social interactions, financial aspects (as gluten-free products tend to be more expensive), and the anxiety experienced by caregivers [21,22,23].

For all of these reasons, a nutrition education program related to CD and GFD should aim at both physical and social health. Moreover, taking into account the high impact of the disease on the people close to the person with CD, as well as the importance of the behaviour of the society for their inclusion, education should be directed not only to the patient but also to the general population [16]. Even if several interventions aimed at the general population have been developed for the prevention and treatment of other diseases with a high prevalence, such as obesity [24,25,26,27], they are much scarcer in the case of CD.

Moreover, food habit interventions have a peculiarity: many of them target children, because it is during childhood that food habits and lifestyles are learned and established, and many of them focus on the school environment [25,26,27,28,29,30,31]. Currently, new educational approaches, such as STEAM, are being introduced in schools. The STEAM curriculum is based on the need to focus on Science (S), Technology (T), Engineering (E), Arts (A) and Mathematics (M) in a transversal and interdisciplinary way in order to transform teaching and learning processes into integrated and creative processes [32]. Moreover, this methodology is now being incorporated into nutrition education programs [33,34].

In view of the above, we present a nutrition education program aimed at increasing the knowledge of the general population in terms of healthy diet, gluten and CD, aiming to help improve the social inclusion and quality of life of people with CD.

## 2. Materials and Methods

### 2.1. Description

The Zeliakide project (the name comes from the union of two Basque words: “zeliako”, which means “celiac”, and “kide”, which means “companion”) is a one-month (4 weeks) intervention program, two-armed cluster, non-randomised and controlled trial, conducted among 10–12-year-old children with a pre- and post-intervention evaluation and 1 month follow-up to assess the effectiveness of the program.

The trial consists of a nutrition education intervention, focused on CD and the GFD. It is an intervention based on competencies and their respective learning outcomes. The teaching methodology chosen is inquiry-based learning (IBL). IBL enables children to acquire skills and knowledge through inquiry. It is a STEAM methodology that seeks to get children to ask questions, inspect, make measurements, design experiments, make logical reasoning based on evidence, and communicate the results.

The intervention is carried out by research experts in nutrition and CD, from the GLUTEN3S research team, and with experience in nutritional education activities. In addition, in order to highlight the role of women in science, they will be mainly women from this group who will go to the classroom to develop the intervention.

### 2.2. Participants

Healthy children in the fifth and sixth years (last cycle) of primary school (10–12 years old) will participate in the study. At this age, they actively work on the concepts of diet and nutrients in the school curriculum. In addition, they also start to work on scientific competence, so it is considered the most suitable time to carry out the intervention. 

Inclusion criteria to participate in the study will be that children study in a primary school that must be used to project based learning and show interest in participating in the project. In addition, schools must have at least one class in the 5th and 6th grades of primary school. The exclusion criterion is school students who have a family or close relationship with any of the GLUTEN3S team researchers.

### 2.3. Sample Size Calculation

Regarding knowledge acquisition, based on the results of a pilot experience of this program as well as other studies found in the bibliography, it has been estimated that to provide a power of 80% and an α error of 5% to detect a small–medium effect size (Cohen’s d of 0.32), 60 participants are required. Since detecting changes in attitude is more complicated, *n* = 120 participants will be proposed for such an outcome. Based in the dropout of similar studies [35,36,37], the final sample size will be increased by 20% to reach 144 children.

### 2.4. Allocation to Groups

**Figure 1:**
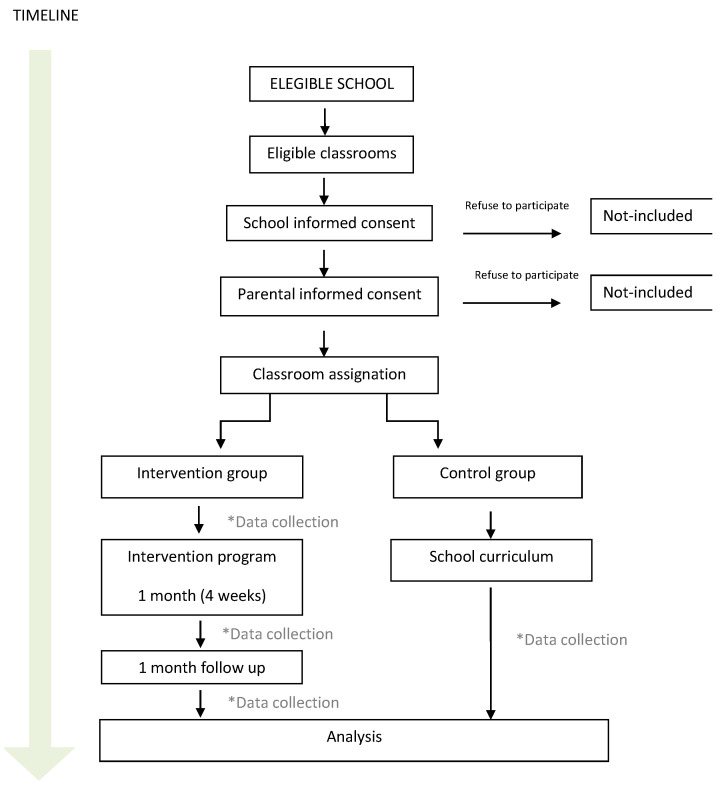
The overview of the Zeliakide intervention study flow. “*Data collection” means the point in time at which data are to be collected.

The distribution of the groups will be based on convenience as judged by experts (see the study flow in Figure 1). The intervention group will be made up of children whose teachers have communicated their wish to actively participate in the program. Teachers need to be involved in the intervention as they are aware of the situation in each classroom and it is necessary to carry out the activities correctly inside and outside the classroom.

As stated, the participants in the study will be students in the last cycle of primary education (10–12 years). The intervention will be carried out with pupils in the first year of this cycle (10–11 years) as, at this point, they acquire knowledge about nutrition and begin to develop scientific competence. All pupils in the same school, academic year and group should be part of the intervention group, since, from an ethical point of view, all groups should acquire the same knowledge during the school year. The control group will be formed by the students in the second year of the last cycle of the same primary school (11–12 years). 

### 2.5. Intervention

#### 2.5.1. Intervention Design

This nutrition education program is adapted to the curriculum of the Spanish schools. The intervention will consist of 8 sessions and the main goals will be (1) to understand the balanced diet and to apply it to one’s own diet, (2) to learn what CD and gluten are, and (3) to promote social inclusion of people with CD.

As aforementioned, it is an intervention based on competencies and respective learning outcomes. Therefore, to design the program, the first step was to define the competencies that children will develop during the intervention (Table 1). These competencies were then organised into learning outcomes, according to Bloom’s degree of cognitive abstraction [38,39] from level 1 to 6, and some activities were designed in order to develop these learning outcomes [40]. 

As mentioned, the teaching methodology chosen to develop learning outcomes is inquiry-based learning (IBL), which has proven to be very effective in science teaching–learning processes [41]. As a result, an inquiry-oriented teaching-learning process was developed, focused on facilitating children’s understanding of the research process. The teaching–learning process will be developed through games and experiments related to the topic of each session, described in Table 1. It must be noted that some topics will be transversally learnt along the intervention. In other words, content from some sessions will influence the achievement of various learning outcomes.

The whole intervention will last 4 weeks; 8 face-to-face sessions (one topic each) will be held during school hours, with 2 sessions per week (Table 2). Each session will last one hour. In addition to the session, each student will have to carry out several tasks at home together with their families, in relation to what they have learnt in class, to reinforce it through family involvement.

The intervention includes activities aimed at encouraging attitudinal and behavioural change related to healthy diet beyond the classroom. Among them, the most important is the “healthy snacks challenge”. This challenge aims to promote healthy mid-morning snacking among pupils. To this end, in the second session of the intervention, students will be divided into groups. Every day (during the month of the intervention), the children will write down their mid-morning snack in a specific section of a workbook. On the last day of each week, the people who are part of the research team will be in charge of giving the corresponding scores for the snacks. Healthy snack, 1 point (fruit, natural dairy products, dried fruit, vegetables, etc.); medium-healthy lunch, 0.5 points (in addition to the aforementioned foods, it includes some unhealthy food); and unhealthy lunch, 0 points (industrial pastries, biscuits, milkshakes or industrial juices, etc.). 

As mentioned above, the children will compete in groups. In this way, collaborative work will be encouraged, where everyone must strive to achieve a common goal. Likewise, every day, they will address the concept of a healthy diet. 

The necessary material for the Zeliakide program has been already designed. A teaching unit has been produced that includes the following material: a notebook containing the contents of the intervention, a virtual classroom where participants can view the contents of each day and interact with the researchers, and the material to carry out the proposed experiments and games (worksheets, power points, laboratory equipment, online games). In order to provide innovative digital material to the classrooms, an augmented reality application that contains all the information in the notebook has also been designed. Through this application, students will be guided by an avatar, a virtual researcher, who will help them carry out the experiments.

#### 2.5.2. Study Procedure

**Figure 2:**
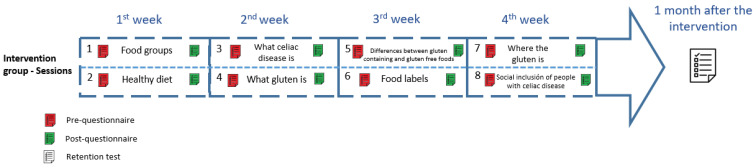
Intervention outline.

The study is schematically depicted in Figure 2. Data will be collected from participants before and after the intervention, using a specific questionnaire designed in order to evaluate the program (Appendix A Table A1). Due to the young age of the children, some limitations when reading and answering a questionnaire are expected, and so they will complete it in parts, thus avoiding the possible discomfort that a long questionnaire may generate in them. The pre-questionnaire part of each session will be completed at the beginning of the corresponding session and the post-questionnaire at the end of this session. They will have to complete a third questionnaire one month after the intervention in order to assess the maintenance of the acquired knowledge and behaviour. 

The pre- and post-intervention questionnaires will be completed with the help of 2–3 members of the research group, in addition to the school responsible teacher. They will help them to understand the questions they have to answer, always avoiding any influence. One month after the intervention, the post-questionnaire will be completed again with the help of the schoolteachers.

The control group will consist of a similar number of students to the intervention group; they will be part of the same school and their socio-demographic characteristics will be similar, even if they will be in the final year of the cycle. They will continue with the normal school curriculum. Students in the control group will be asked to complete a questionnaire as the intervention group, and they will have to answer it in the same month in which the intervention of the other group is being carried out. They will be helped by their teachers to understand the questions if necessary and will have to complete it in one session, since the aim is to avoid altering the usual routine of their classes.

Upon completion of the intervention, parents or guardians will be requested to fill out an online questionnaire to gather their feedback on the project as well as their children’s opinions, as reported by the parents.

### 2.6. Outcome Measures

#### 2.6.1. Primary Outcomes

Due to the fact that the present intervention is based on competencies and learning outcomes, the results are also proposed according to them, differentiating between knowledge-related results and those related to behaviour, which are more difficult to achieve. The primary outcomes are shown in Table 3. 

As mentioned, the primary outcomes will be measured by a pre- and a postquestionnaire reported by children at the beginning of the session, before the activities and discussion of the topic, and at the end of the session. They will be measured again one month after the intervention.

#### 2.6.2. Secondary Outcomes

The secondary outcomes will be measured immediately after completion of the whole intervention. The secondary outcomes are as follows:

- Opinion of the parents/guardians of the children in relation to the project: it will be completed by parents/guardians, online and without the presence of any member of the research group.
- Opinion of the children about the project reported by their parents/guardians: it will be completed by parents/guardians, online and without the presence of any member of the research group.

### 2.7. Data Management and Monitoring

Data will be stored in an anonymised form on corporate devices where only participating researchers will have access to credentials. All data provided for this research will be confidential as required by the European Data Protection Regulation [42] (EU2016/679). Data will be kept as long as their deletion is not requested by the person concerned and, in any case, as long as the periods of appeal and/or appropriate claim are open or as long as they continue to respond to the purpose for which they were obtained. The data controller is the University of the Basque Country (HN.GLUTEN3S, TI0233). 

### 2.8. Statistical Analysis

The description of the characteristics of the participants and data from the pre-, post- and retention test (one month after) questionnaires will be reported per group (control and intervention). 

For the analysis of the differences between groups, a variety of statistical tests will be used depending on the type of response to the questions (continuous, discrete or qualitative). Based on the distribution of the variables of interest in the sample, descriptive statistics for continuous data will be presented using the mean and standard deviation or the median and interquartile range. Qualitative data shall be presented as frequencies and percentages. Quantitative variables will be analysed using an ANOVA of repeated measures test using the group as a factor of the independent level (parametric) or Wilcoxon’s test segmenting by group (non-parametric). On the other hand, qualitative variables in two categories will be analysed with Chi-square and Cochran’s Q tests. Finally, qualitative variables in more than two categories will be analysed with Chi-square and McNemar Bowker’s tests.

Data will be entered and analysed using SPSS 28.0 software. A confidence interval of 95% and significance level of *p* < 0.05 will be assumed.

## 3. Discussion

So far, nutrition education about CD and diet has been focused on people suffering from CD [43,44,45,46,47] as it is necessary to improve their health and quality of life. Nevertheless, nutrition education should go beyond the patient, since family members and social environments also have an influence on the management of the disease, as previous studies have shown [48,49,50,51,52,53,54,55].

Moreover, as people with CD reported, one of the issues affecting their quality of life is their social activity [56]. These people as well as people in charge of them suffer anxiety and depression, partly due to negative feelings of bothering other people and being excluded from social activities [13,57]. Adolescents with CD describe difficulties in having confidence in other people because those people often do not know, do not understand or do not care enough about their dietary needs [58]. This likely happens because of a lack of awareness, which leads to a lack of empathy in people surrounding them. Therefore, it is very interesting to consider this aspect in the education of the general population, linked to the healthy diet learning process. 

The nutrition education programs carried out so far in schools have been mostly focused on improving the general diet and do not consider the overall situation of a non-insignificant part of the population, such as people going on a GFD. The Zeliakide program will be developed in schools because it is a trusted place for children and is where they spend most of their time [59,60]. It is their usual learning environment and is considered a suitable place for this kind of intervention [25,26,27,28,29,30,31,61,62]. 

Regarding the methodology of the program, the inquiry-based learning methodology has been chosen, where children learn by doing [32]. This methodology is well known and proven to be effective and encourages interaction and reflection among the children [63,64,65]. Many nutrition education programs focus on social cognitive theory [66,67,68,69]. However, in this case, the program goes further by adapting a methodology focused on teaching science, as one of the main goals of the curriculum of primary children is get closer to STEAM education. Moreover, the Zeliakide programme will be implemented by the Gluten3S research team, and scientists will go to the schools to explain, first hand, how to make the proposed experiments and interact with the children in the development of scientific reasoning. 

On the other hand, there is increasing concern among policy-makers and practitioners about the under-representation of girls and women in STEAM education [70]. Indeed, Kim et al. conducted a study that demonstrated how perceptions regarding the inclusion or exclusion of individuals in STEAM fields can be altered through intervention and educational initiatives [71]. In the present protocol, a female scientist will implement the programme and work in situ with children in the classroom. 

Competences and learning outcomes were carefully established prior to the design of the programme according to Bloom’s taxonomy, based on the level of cognitive abstraction [39,40]. The higher the level, the more complex the learning becomes. Subsequently, the activities were designed based on the IBL methodology, and the evaluation method for each learning outcome was defined. The wording of each question was carefully selected, with the help of the teachers involved in the project, to fit the cognitive level of children. In addition, the activities were designed to drive a measurable change in attitude towards a healthy diet in general and towards people with CD in particular. This careful procedure allows the intended objectives and measure outcomes to be effectively achieved with robustness. Therefore, the results obtained from the application of this protocol will accurately assess the changes in the knowledge and attitude of the participants. In addition, it is expected to indicate which aspects of those studied in the programme should be further developed in schools.

The design of the study also took into account the results obtained in a previous pilot work conducted by the Gluten3S research team [40,65]. This procedure is fundamental to accurately estimate the required sample size and avoid involving an excessive or insufficient number of participants, ensuring that the intervention’s effect can be properly observed. Thus, it has been estimated that a total of 144 children should be included in the study in order to detect a small-to-medium increase in knowledge, such as an increase in approximately one point on a scale of 0–4; thus, a sample size of this magnitude is necessary. 

The teaching unit is a complete set of materials, such as a student’s and a teacher’s notebook with all the activities explained in detail and various games, especially designed for the Zeliakide Programme. It also includes an innovative augmented reality application to reinforce the use of IBL methodology. It is considered that this material may be useful for future interventions or didactic activities, and it will be freely available and accessible to anyone who wants to use it, upon request. Moreover, it can be easily used autonomously by teachers and trainers. 

Zeliakide does not forget the importance of family participation in nutritional education activities, since results of a meta-analysis revealed that interventions that included family members produced larger effects than interventions focusing only on children [61,72,73,74]. Therefore, the intervention presented here includes activities to be carried out at home to involve children’s parents and guardians. Besides that, two short sessions (60 min) per week will be held at school over a period of one month. It must be highlighted that it seems to be appropriate to transmit the information in small doses and spread out over time for optimal learning [29,72,75,76,77].

The next step will be to implement and measure the effectiveness of the intervention proposed here. This step will be taken in the near future. It is important to highlight that the effectiveness of the intervention will be assessed by monitoring the participants using a questionnaire one month later. This approach takes into consideration the findings of previous studies that emphasise the significance of evaluating the long-term results. It is crucial to ensure that the acquired knowledge and behaviour can be sustained over time, thus providing a more comprehensive understanding of the intervention’s impact [77,78,79].

In this sense, it should be noted that this study has some weaknesses. It is a non-randomised trial; thus, some bias is expected. In addition, the control group attends a higher level at the same school than the intervention group, so there could be differences in knowledge and attitudes due to age. However, it has been considered that the control group would be even more different by choosing control groups of the same age but from different schools, since socio-cultural differences by territory (and consequently, by school) are an important aspect to take into account. At the same time, this weakness could be considered a kind of strength, because most interventions at schools do not consider any control group at all [80]. It is also remarkable that the design of Zeliakide is practical and suited to the reality of schools, and so it is easily applicable to other centres in the future. 

## 4. Conclusions

The treatment of CD extends beyond dietary measures. Thus, it is now acknowledged that the psychological and social aspects of the disease are equally important. Ensuring the social inclusion of individuals with CD in society is crucial. Therefore, Zeliakide aims to make a societal impact by targeting a significant group: schoolchildren. It is important to note that this protocol has been carefully designed. It is based on well predefined competences and learning outcomes, which are developed through a collaborative-active methodology and are assessed precisely using a specific evaluation tool.

It is anticipated that Zeliakide will be seamlessly integrated into the school curriculum, enhance STEAM competence among children, improve their understanding of balanced and gluten-free diets, and increase knowledge about CD. Additionally, it is expected to foster positive attitude changes towards this topic.

## Figures and Tables

**Table 1:** Competency-based intervention: Zeliakide description.

Competence	Learning Outcome	Activity	Topic Day
The student should be able to place the food groups in the food pyramid.	*Bloom level 3. Application* The student adequately organises different foods in the pyramid.	Game about the food pyramid.	Day 1. Food groups Day 2. Healthy diet
The student should be able to identify dietary errors and make recommendations for its modification.	*Bloom level 4. Analysis* The student identifies foods to be consumed infrequently. *Bloom level 6. Evaluation* The student offers recommendations to adapt its consumption.	Complete and adapt a 24 h recall.	Day 1. Food groups Day 2. Healthy diet
The student should be able to consume less food from the top of the pyramid.	*Bloom level 6. Evaluation* Students consume less unhealthy food than before the activities.	Game about the food pyramid. Complete and adapt a 24 h recall. Healthy snacks challenge.	Days 2 to 8. Healthy snacks challenge. Day 2. Healthy diet (transversally)
The student should be able to explain what celiac disease is.	*Bloom level 2. Understanding* The student acknowledges that gluten harms people with celiac disease and makes them sick. The student concludes that people with CD should follow a gluten-free diet.	Game to learn the characteristics of the disease.	Day 3. Celiac Disease
The student should be able to analyse gluten	*Bloom level 3. Application* The student manipulates gluten and finds it. The student figures out that gluten gives elasticity to the doughs. *Bloom level 4. Analysis* Students deduce where gluten is by analysing food labels.	Experiment 1: Learning by doing: pasta made with different types of flour. Experiment 2: Food analysis through the senses.	Day 4. What gluten is Day 5. Where gluten is (transversally)
The student should be able to classify food groups according to gluten content.	*Bloom level 4. Analysis* The student identifies the food groups that contain gluten: placing them in cereals. The student concludes that processed foods may contain gluten.	Experiment 3: Gluten detection experiment, immunochromatography. Experiment 4: Study of food labelling.	Day 6. Food labels Day 7.Where gluten is Day 4. What gluten is (transversally)
The student should be able to understand the work of scientists, to know that the gluten content of foods is analysed through experimentation.	*Bloom level 6. Evaluation* The student is able to perform experimental work, observe, analyse and evaluate the results.	All the experiments.	Day 4. What gluten is Day 5. Differences between gluten and gluten free foods Day 6. Food labels Day 7. Where gluten is
The student should be able to assess the impact of own actions on others.	*Bloom level 6. Evaluation* The student demonstrates empathetic behaviour. The student selects actions and behaviours to overcome differences between people.	Game to learn the characteristics of the disease. Case study to work on inclusion: discussion.	Day 8. Social inclusion of people with celiac disease Day 3. Celiac Disease (transversally) Day 5. Differences between gluten and gluten free foods (transversally)

Meaning of “transversally”: some activities or days, in addition to developing their specific competencies and learning outcomes, contribute in a transversal way in the achievement of others. Thus, “transversal” has been used to indicate this situation.

**Table 2:** Zeliakide intervention study schedule.

Intervention Group				Control Group
**Week**	**Topic Session 1**	**Topic Session 2**	**Challenge**	No intervention
1	Day 1. Food groups	Day 2. Healthy diet	“Healthy snacks challenge”	
2	Day 3. Celiac Disease	Day 4. What gluten is		
3	Day 5. Differences between gluten containing and gluten free foods	Day 6. Food labels		
4	Day 7. Where gluten is	Day 8. Social inclusion of people with celiac disease		

**Table 3:** Competency-based intervention: outcome measures.

Competence	Outcome Measures
The student should be able to place the food groups in the food pyramid.	Change in nutrition knowledge
The student should be able to identify dietary errors and make recommendations for modification.	Change in nutrition knowledge
The student should be able to consume less food from the top of the pyramid.	Change in behaviour in relation to the consumption of unhealthy foods
The student should be able to explain what celiac disease is.	Change in Celiac Disease knowledge
The student is able to assess the impact of their own actions on others.	Change in intentional behaviour related to the social inclusion of people with celiac condition: perception of the influence of one’s own actions on others.
The student should be able to analyse gluten	Change in knowledge regarding gluten and its presence in foods
The student should be able to classify food groups according to gluten content.	Change in knowledge regarding gluten and its presence in foods
The student should be able to understand the work of scientists, to know that the gluten content of foods is analysed through experimentation.	Change in attitude in relation to interest in science

## Data Availability

Data are contained within the article and Supplementary Materials.

## Supplementary Materials

The following supporting information can be downloaded at: https://www.mdpi.com/article/10.3390/nu16030338/s1, File S1: Request for informed consent for research.

## Appendix A

**Table A1:** Questionnaire to be completed by children to evaluate the intervention.

Competence	Question		Response
1	Do you know which foods are found in the different parts of the food pyramid?		Yes/No/No response
	Which foods are at the top of the pyramid?		Open question
	Which foods are at the bottom of the pyramid?		Open question
	Of the food pairs below, which do you think is highest in the food pyramid?		Tomato/Sweet, Pizza/Rice, Bread/Juice, Olive oil/Red meat, Potato/Ham
2	Of the foods you eat during the day, do you know which are unhealthy?		Yes/No
	Which are the unhealthy foods you eat?		Open question
	Are the foods that are often featured in advertisements usually healthy?		Yes/No/There is a bit of everything
	How much fruit and vegetables should you eat per day?		Open question
	What do you think you should improve your diet, for example, by eating more fruit, less sweets?		Open question
3	How many days a week do you usually take a healthy lunch to school (fruit, dairy products, wholemeal bread…)?		Scale 0 days–5 days
	Of the foods at the top of the food pyramid, how many do you eat at the weekend?		I eat them on Fridays, Saturdays and Sundays, more than once a day. I eat them on Fridays, Saturdays and Sundays, once a day. I only eat them one day at the weekend. I don’t eat them every weekend, only once in a while.
	Do you think it is important to eat a healthy diet?		No, if you do sport it is enough Yes, it is important but it is better for everyone to eat what they want Yes, it is very important for me.
	With what you have learned in these sessions, do you think you are able to improve your diet?		Yes, I know what mistakes I make in my diet and I am able to improve it Yes, I have learned a lot but I am not able to improve my diet No, I have not learned enough
	In the last month, have you eaten more, less or the same amount of the following food groups? (dairy products, fruits, vegetables, meat, fish, pulses, cereals, sweets)		Less/Equal/More/I do not know/No response
4	How much do you know about CD?		Scale 0 (nothing)–4 (much)
	What symptoms do people with CD have?		Open question
	When do people with CD develop symptoms?		Open question
	What compound in food is harmful to people with CD?		Open question
5	How do you think people with CD feel?		Open question
	Does a person with CD enjoy eating?		Scale 0 (nothing)–4 (much)
	What would you do if it was your birthday and a classmate had CD?		Bring the cake that I like the most. Bring the cake that I like the most and another gluten-free cake for the person with CD. Bring a gluten-free cake for everyone. No response.
6	Where is the gluten?		Open question
	What makes a dough elastic?		Gluten Water I do not know
	How can we know (without experimenting) whether a food contains gluten or not?		Open question
	Which symbols indicate that a food does not contain gluten?		Open question
7	Which of the following foods a person with CD cannot eat?		Meat and fish Eggs and dairy Fruit and vegetables Wheat-based products: biscuits, bread and pasta Rice and maize Sweets They can eat anything I do not know
	From the following list of foods, which ones may contain gluten?		Raw rice, raw beans, fresh fruits, fresh vegetables, natural dairy products, fresh meat or fish, biscuits, some sweets, sweetened cocoa powder, some ice-creams, chocolate
	Can a prepared food (cream of vegetables, prepared beans…) have gluten, even if it is made from gluten-free ingredients?		Yes/No
8	Please rate these sentences according to your level of agreement.	In order to detect gluten, laboratory experiments must be carried out.	Scale 1 (not agree)–5 (very agree)
		It is important for scientists to do experiments.	Scale 1 (not agree)–5 (very agree)
		In the future I would like to become a scientist.	Scale 1 (not agree)–5 (very agree)
		Researchers are crazy, weird and male.	Scale 1 (not agree)–5 (very agree)
