# Supplementary material for: Study Protocol for a Controlled Trial of Nutrition Education Intervention about Celiac Disease in Primary School: ZELIAKIDE Project
Source: Nutrients. 2024 Jan 23;16(3):338. doi: 10.3390/nu16030338 (PMC10857138; doi:10.3390/nu16030338)
Supplement: Supplementary file 1 [file nutrients-16-00338-s001.zip › nutrients-2814965-supplementary.pdf]

eman ta zabal zazu

Universidad del País Vasco — Euskal Herriko Unibertsitatea

REQUEST FOR INFORMED CONSENT FOR RESEARCH

We are contacting you owing to a project we are developing:

Project: Nutrition Education: Safe, Balanced and Inclusive Gluten-Free Diet

Research Group: GLUTEN3S, UPV/EHU

What is the aim of the project?

The overall aim of this project is to promote the physical and social health and quality of life of the population with celiac disease through education of the general population. In addition, it seeks to promote a healthy and balanced diet in the general population.

Description of the project, method used:

The GLUTEN3S Research Group (UPV/EHU) conducted multiple studies on the gluten-free diet of individuals with celiac disease in the Basque Country. Based on their findings, the group has recommended dietary guidelines to address common errors. These guidelines include increasing the intake of dietary fiber and complex carbohydrates while reducing the consumption of saturated fats and proteins. To achieve this, they suggest increasing the consumption of gluten-free cereals, legumes, fruits, vegetables, and greens, while decreasing the consumption of fatty meats. It is also considered necessary to increase information about: gluten, celiac disease and balanced gluten-free diet (socially and nutritionally).

The methodological approach we have created is centered around developing and implementing activities as part of the "Nutrition Education: Safe, Balanced and Inclusive Gluten-Free Diet" project. These activities aim to provide guidance and promote a gluten-free diet that is safe, balanced, and inclusive.

A didactic unit on celiac disease has been designed for the development of scientific competence in the primary school classroom. In order to test its effectiveness, the aim is to implement the didactic activities in the classroom.

Type of test:

The trial consists of 8 one-hour sessions that will take place over one month. The sessions will include games, experiments and discussions on healthy and balanced diet, celiac disease and gluten-free diet.

Participants will be asked to fill in a total of 3 simple surveys, the first one before the workshop, and the second and third after the workshop. The aim of these surveys is to evaluate and improve the activity based on the degree of learning and the opinion of the participants. Parents or guardians of participants will be asked to complete an online questionnaire at the end of the intervention at the school.

Participants are requested to:

1) Attend the sessions

2) Complete the aforementioned surveys

Therefore, express authorisation and consent is requested from the parents or guardians of the participants, as indicated in the data protection regulations, for their participation in this project and for the subsequent dissemination and publication of its results, in a general and completely anonymous way. Participation in the study does not involve any risk for the participant, the only risk associated will be the use of their time.

Participation in the study by completing the surveys is voluntary. Refusal to participate is not prejudicial. Videos and photos will be taken during the activities. Likewise, the participant may leave the study at any time without any prejudice or action against him/her. Participation is altruistic and no remuneration will be received for it.

The general results of the activity will be made known through communications to national or international congresses and/or publications in innovation research journals.

If you would like to obtain additional information or clarify any doubts, please call XXXXX (Contact person: Maialen Vazquez). If you agree with your child's participation, please sign the following informed consent form.

DECLARATION OF INFORMED CONSENT

I have read the information sheet provided to me and understand what it says. I understand that participation in the study is voluntary, that I may withdraw at any time without prejudice of any kind, and I agree to participate.

I know that this study is part of the project "Nutritional Education: Safe, Balanced and Inclusive Gluten-Free Diet".

Likewise, I accept that the global results obtained from this activity will be disseminated and communicated through congresses and scientific publications. The dissemination and publication of the results of the project will be done in a general way, no individual or personal data will be disclosed.

I GIVE MY CONSENT:

a) To participate in the research, through the completion of 3 surveys about the contents covered in the workshop by my son/daughter and a questionnaire by me.

YES...................NO............

NAME AND SURNAME OF THE PERSON ATTENDING

………

NAME AND SURNAME OF MOTHER/FATHER/LEGAL GUARDIAN (1)

………

MOTHER/FATHER/LEGAL GUARDIAN ID (1)

………

Signature of the person collecting the consent

Signature of reporting person

Sgd: ………………………………………………………………………

Sgd:…………………………………………………………

Date:…………………………………………………………………

ACCEPTANCE OF DATA USAGE

(Name)..., I agree that the data obtained in the study about "Nutrition Education: Safe, Balanced and Inclusive Gluten-Free Diet" can be used to analyse the effectiveness of the workshop and to achieve a future improvement of it.

I understand that my data will be treated with confidentiality and respecting anonymity, as required by the rules of data protection and confidentiality (Organic Law 15/1999 and 41/2002).

Permission to keep data: YES (I accept)......... NO (I do not accept) ....

Signature of the interested individual

…………………………………………………………….

ACCEPTANCE OF THE USE OF AUDIOVISUAL MATERIAL

(Name)..., I agree that the photographs or videos taken in the workshop about "Nutrition Education: Safe, Balanced and Inclusive Gluten-Free Diet" can be used for the dissemination of the workshop only in congresses or scientific publications.

I understand that my data will be treated with confidentiality and respecting anonymity, as required by the rules of data protection and confidentiality (Organic Law 15/1999 and 41/2002).

Permission to keep data: YES (I accept)......... NO (I do not accept) ....

Signature of the interested individual

....

*All data you have provided to us for this research is confidential. We will protect it as required by the European Data Protection Regulation (EU2016/679) and will only use it for the research we have explained

to you. If you would like to consult or modify them, or have them deleted or not used for any of the research purposes, please contact us at the following address:

- The code of the data processing is: TI0233

- The name of the data processing is: HN.GLUTEN3S.

- The purpose of this processing is: To test the effectiveness of a nutrition education program.

- The data controller is the University of the Basque Country (UPV/EHU).

Identity: University of the Basque Country / Euskal Herriko Unibertsitatea
TAX ID: Q4818001B
Postal address: Barrio Sarriena s/n, 48940-Leioa (Bizkaia)
Website: www.ehu.eus
Contact details of the Data Protection Delegate: dpd@ehu.eus

- The personal data requested are: age, gender, school and grade, parents' employment, whether or not they suffer from celiac disease and their opinion regarding the topics covered.

- The period of conservation of your data will be: The data will be kept as long as their deletion is not requested by the person concerned and, in any case, as long as the periods of appeal and/or appropriate claim are open or as long as they continue to respond to the purpose for which they were obtained.

- The legitimacy of the treatment is: your informed consent.

- Transfers: no transfers will be made except by legal mandate.

- International transfers of your data: No international transfers will be made.

- The rights over your data are those of access, deletion, rectification, opposition, limitation of processing, portability and oblivion. You can exercise them by sending your request to dpd@ehu.eus.

- Additional information is available at http://www.ehu.eus/babestu

- Complete information about this processing is available at: https://www.ehu.eus/es/web/idazkaritza-nagusia/ikerketa-datu-pertsonalen-tratamenduak

Contact: Maialen Vazquez. University of the Basque Country, UPV/EHU, Department of Pharmacy and Food Sciences

E-mail: xxxxxxxxxxx
